# Expression of PD-1 on CD4^+^ T cells in peripheral blood associates with poor clinical outcome in non-small cell lung cancer

**DOI:** 10.18632/oncotarget.9316

**Published:** 2016-05-12

**Authors:** Hong Zheng, Xin Liu, Jianhong Zhang, Shawn J. Rice, Matthias Wagman, Yaxian Kong, Liuluan Zhu, Junjia Zhu, Monika Joshi, Chandra P. Belani

**Affiliations:** ^1^ Penn State Hershey Cancer Institute, Penn State College of Medicine, Hershey, PA, USA; ^2^ Institute of Infectious Diseases, Beijing Ditan Hospital, Capital Medical University, Beijing Key Laboratory of Emerging Infectious Diseases, Beijing, China

**Keywords:** PD-1, peripheral blood, T cells, prognosis, NSCLC

## Abstract

Recent success of using agents inhibiting the major immune check point, programmed cell death-1 (PD-1) pathway, offers a great promise for effective cancer therapy. Two blocking antibodies for PD-1, nivolumab and pembrolizumab have recently been approved for treating advanced recurrent non-small cell lung cancer (NSCLC). Activation of PD-1 on T cells and PD-L1 on tumor cells or antigen presenting cells leads to T cell exhaustion and ultimately tumor growth. In this study, we performed flow cytometry analysis of peripheral blood samples collected from patients with advanced NSCLC at initial diagnosis. We report that surface expression of PD-1 on CD4^+^ T cells has a prognostic value in NSCLC patients, as high expression of PD-1 is associated with a shorter progression-free survival and overall survival. Importantly, we also found that high PD-1 expression on peripheral CD4^+^ T cells is associated with inferior clinical response in a subset of patients who received anti-PD-L1 treatment, indicating a potential predictive value of this marker. This work highlights the potential of a non-invasive and effective method to determine prognostic and predictive biomarkers for inhibiting the PD-1 pathway in NSCLC patients.

## INTRODUCTION

Lung cancer remains the leading cause of cancer-related death in the United States, with approximately 160,000 deaths every year. The majority of these patients have non-small cell lung cancer (NSCLC). The prognosis for metastatic NSCLC is generally poor with the overall five-year survival of < 5% [1]. Platinum-based chemotherapy has been the standard treatment for advanced NSCLC in addition to targeting patient-specific tumor driving mutations (i.e. EGFR) [2, 3]. These therapies, however, are limited by significant toxicities and development of resistance. Furthermore, only a fraction of these patients have targetable mutations in their tumor.

The recent success with using reagents targeting negative regulators of the immune checkpoint proteins PD-1/PD-L1 offers great promise for effective cancer therapy [4, 5, 6, 7, 8, 9]. Programmed cell death-1 (PD-1) pathway is a major immune checkpoint [10]. The interactions between PD-1 on T cells and PD ligand-1 (PD-L1) on tumor cells or antigen presenting cells (APCs) mutes T cell activation and T-cell-mediated tumor cell killing [11, 12, 13, 14]. Agents blocking the PD-1 pathway, nivolumab and pembrolizumab, have recently been approved by the FDA for treating several solid tumors including advanced NSCLC. Antibodies blocking PD-L1 have also demonstrated clinical efficacy in patients with NSCLC [15].

It is important to identify prognostic and predictive biomarkers associated with PD-1 pathway inhibitors to guide clinical decisions. Much attention has been focused on testing PD-1 or PD-L1 expression in tumor tissues, which is challenging due to the difficulties in obtaining tissue samples and the lack of consensus among the available antibodies for immunohistochemistry staining [16, 17, 18, 19]. Here we analyzed blood samples collected from a cohort of 42 patients with advanced stage NSCLC. We utilized flow cytometry to evaluate peripheral expression of PD-1 and further dissect their prognostic and predictive value in NSCLC.

## RESULTS

### PD-1 is elevated on CD4^+^ T cells in peripheral blood of patients with NSCLC

**Figure 1:**
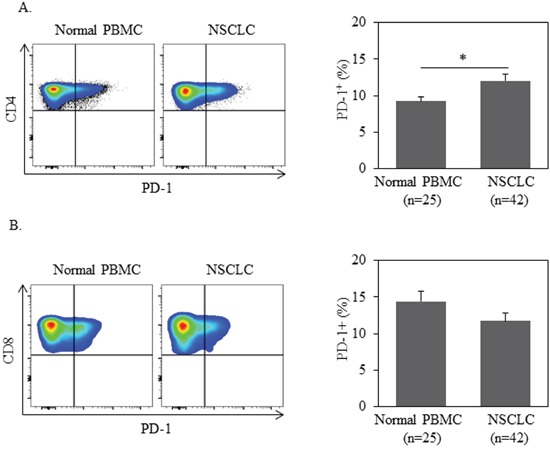
PD-1 expression in CD4+ T cells is higher in NSCLC patients than ones in normal donors 1 million of PBMC from patients or normal donors were used for quantification of PD-1+% in CD3+CD4+ or CD3+CD8+ T cells by flow cytometry. Statistical significance was determined by a Student t test (*, *p*<0.05).

We assessed PBMCs from 42 NSCLC patients at initial diagnosis for PD-1 expression on both CD4^+^ and CD8^+^ T cells. PBMCs from 25 healthy donors (HD) were tested as controls. All 42 patients evaluated in our study were diagnosed with advanced stage (stage IIIB or IV) NSCLC (Table 1). As shown in Figure 1A–1B, the frequency of PD-1^+^ cells among CD4^+^ T cells from NSCLC patients was significantly higher than those from HD (mean frequency 13.3%±1.1% *vs*. 8.8%±0.7%, *P*=0.0045). In contrast, expression of PD-1 on CD8^+^ T cells was comparable between NSCLC patients and HD. The data suggest that CD4^+^, and not CD8^+^, T cells play a pivotal role in PD-1-dependent immune tolerance in NSCLC patients.

**Table 1:** Characteristics of 42 advanced non-small cell lung cancer patients

	CD4+ PD-1 % high	CD4+ PD-1 % low	P values	Statistical Test
Age (years)	Median 65	Median 62	0.371	Mann_Whitney U
Gender				
Male	5	14	0.337	Fisher's
Female	10	13	0.337	Fisher's
Stage				
IIIB	2	2	0.608	Fisher's
IV	13	25	0.608	Fisher's
Histology				
Adenocarcinoma	13	20	0.451	Fisher's
SCC	2	7	0.451	Fisher's
Smoking status				
Smoker	11	24	0.225	Fisher's
Non-smoker	4	3	0.225	Fisher's
Treatments				
Chemotherapy	11	16	0.569	Fisher's
Immunotherapy	2	3	0.569	Fisher's
Targeted therapy	2	8	0.569	Fisher's
**Total**	**15**	**27**		

### Tumor burden is independent of PD-1^+^ T cells from peripheral blood in patients with NSCLC

**Figure 2:**
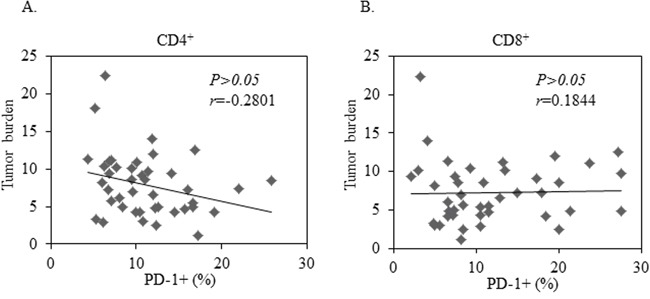
No Correlation between tumor burden and PD-1 expression in CD4+ or CD8+ T cells in 42 patients with advanced NSCLC A least-squares linear fit is shown as a thick black line. Statistical significance was determined by a Pearson Correlation (*p*>0.05).

We next assessed the correlation of PD-1 expression with clinical characteristics of NSCLC patients. Greater tumor burden is considered a negative impact on cancer prognosis. We analyzed the correlation between PD-1 expression on peripheral T cells and tumor size in our patients with NSCLC. There was no significant correlation between PD-1 expression on CD8+ T cells and tumor burden (Figure 2B, *P*=0.450). There was a positive correlation among patients with higher PD-1 expression on CD4^+^ T cells and the presence of smaller tumor burden, but this did not reach statistical significance: (Figure 2A, *P*=0.861). Consistently, no correlation was found between the PD-1 expression on peripheral blood T cells and the stage of NSCLC patients (data not shown).

### Expression of PD-1 on T cells from peripheral blood does not correlate with PD-L1 expression on tumor tissues in patients with NSCLC

**Figure 3:**
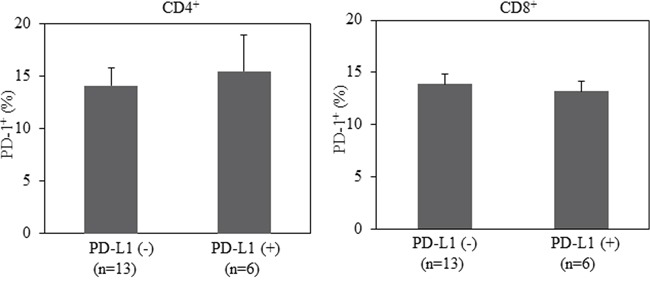
No correlation between PD-L1 expression in lung cancer tissues and PD-1 expression in CD4+ or CD8+T cells from patients with advanced NSCLC Statistical significance was determined by a Student t test (*p*>0.05).

Although controversial, a large number of studies have demonstrated that PD-L1 expression on tumor tissues predicts clinical response to inhibitors of the PD-1 pathway. Among 42 NSCLC patients evaluated in our study, 19 patients were tested for PD-L1 expression by immunohistochemistry (IHC) in their tumor tissues. We further assessed whether the level of PD-1 expression on peripheral blood T cells associates with PD-L1 in tumor tissues. As shown is Figure 3, the percentage of PD-1 expression on T cells is comparable between PD-L1^−^ and PD-L1^+^ population. This occurs in both CD4^+^ and CD8^+^ T cells. Thus, the expression of PD-1 on T cells from peripheral blood does not associate with PD-L1 expression on tumor tissues in patients with advanced NSCLC.

### High expression of PD-1 on CD4^+^ T cells at initial diagnosis associates with poor clinical outcome in NSCLC

**Figure 4:**
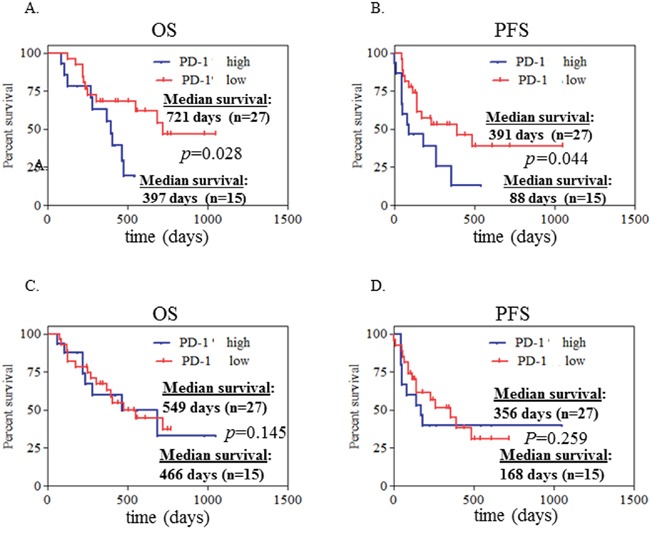
High expression of PD-1 on CD4+ T cells at initial diagnosis associated with poor clinical outcome in advanced NSCLC patients Kaplan-Meier curves of 42 patients with advanced NSCLC segregated into 2 groups **A, B.** according to the median value of PD-1+ CD4+% cells (with 12.27% as a threshold). PD-1+% CD4+ cells >12.27% was defined as PD-1+% high while <12.27% as PD-1+% low. Likewise, these 42 patients were also segregated into 2 groups **C, D.** according to the median value of PD-1+ CD8+% cells (with 11.75% as a threshold). PD-1+% CD8+ cells >11.75% was defined as PD-1+% high while <11.75% as PD-1+% low. Statistical significance was determined by a Log-rank (Mantel-Cox) Test.

**Figure 5:**
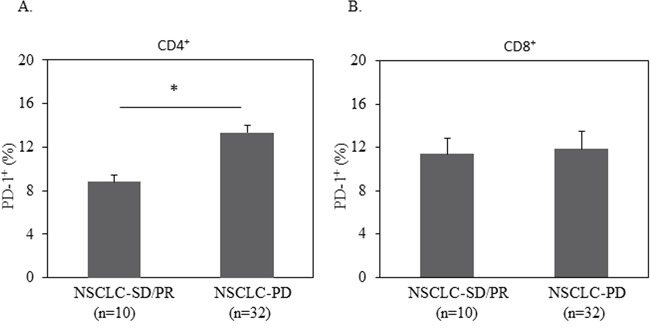
Elevated PD-1 expression on CD4+ T cells was correlated with disease progression 1 million of PBMC from patients with progressed disease (PD) or stable disease (SD) or partial response (PR) were used for quantification of PD-1+% in CD3+CD4+ or CD3+CD8+ T cells by flow cytometry. Statistical significance was determined by a Student t test (*, *p*<0.05).

Based on the level of PD-1 expression on CD4^+^ T cells, we defined high-PD-1 (PD-1>12.27%) vs. low-PD-1 (PD-1<12.27%) subgroups in NSCLC patients. The mean value (12.27%) of PD-1 expression on CD4^+^ T cells of the 42 NSCLC patients was the cutoff. Seventeen patients are classified in the high-PD-1 group and 25 in the low-PD-1 group. We analyzed clinical characteristics and found no significant difference between the two groups in terms of age, gender, histology, stage, smoking history, and treatments (chemotherapy, targeted-therapy or immunotherapy). We next investigated PD-1 expression on clinical outcome. We found a significantly lower rate of both overall survival (OS) and progression free survival (PFS) in the high-PD-1 group compared with the low-PD-1 group (median OS: 397 days vs. 721 days, *P*=0.028, Figure 4A; median PFS: 88 days vs. 391 days, *P*=0.044, Figure 4B). Consistent with this finding, elevated PD-1 expression on CD4^+^ T cells was observed in patients with early progression of disease, compared with patients who had partial response and stable disease (Figure 5). These data demonstrate that PD-1 expression on peripheral CD4^+^ T cells is associated with worse clinical outcomes in NSCLC, independent of age, stage, and histology type. In addition, the same analyses were performed for CD8^+^ T cells and no correlation between PD-1 expression on peripheral CD8^+^ T cells and clinical outcomes was observed (Figure 4C–4D, Figure 5B).

### High expression of PD-1 on CD4^+^ T cells at initial diagnosis associates with poor clinical outcome after anti-PD-L1 treatment

Six patient tumors were found to be PD-L1 positive by immunohistochemistry. Five of these patients were treated with a PD-L1 inhibitor while one patient received chemotherapy. We further dissected the association of PD-1 expression on CD4^+^ T cells from peripheral blood to clinical outcome. Among the three patients who had low PD-1 expression at initial diagnosis, two had stable disease; one had disease progression at day 264. All three are alive at the last follow-up (day 645, 539, and 461 respectively). In contrast, all three patients who had high PD-1 expression had disease progression (at day 52, 43, and 120 respectively), two of whom had died at day 268 and 466 (Table 2). Although our conclusion is limited due to the small sample size, we see a trend that high PD-1 expression on CD4^+^ T cells from peripheral blood associates with poor clinical outcome after treatment with anti-PD-L1 antibody, indicating a potential value of this marker in prediction of response to PD-1 pathway inhibitors.

**Table 2:** Characteristics of 6 advanced NSCLC patients with positive PDL-1

Patient #	Patient #1	Patient #2	Patient #3	Patient #4	Patient #5	Patient #6
age	66	67	65	77	55	57
gender	female	female	female	female	female	female
histology	ACA	ACA	SCC	ACA	ACA	ACA
Smoking status	smoker	smoker	smoker	Non-smoker	Non-smoker	smoker
PD-1% CD4+	6.12	12.18	9.99	25.89	15.73	25.53
last follow-up	645 days with stable disease	539 days with stable disease	264 days with progressed disease	43 days died with progressed disease	120 days with progressed disease	52 days died with progressed disease

Note: ACA is abbreviation for adenocarcinoma, SCC is abbreviation for squamous cell carcinoma.

## DISCUSSION

In this study, we evaluated the phenotype of peripheral blood T cells collected from NSCLC patients and determined the correlation between PD-1 expression on T cell of peripheral blood with clinical outcome. The three most important findings are: 1) PD-1 expression on CD4^+^ T cells is significantly elevated in patients with NSCLC at initial diagnosis, compared with those of healthy donors. 2) High expression of PD-1 on CD4^+^ T cells is strongly associated with poor clinical outcome manifested by significant shorter OS and PFS in patient with NSCLC and this is prognostic. 3) PD-1 expression on CD4^+^ T cells has potential predictive value in NSCLC as high PD-1 expression associates with worse clinical outcome after anti-PD-L1 treatment.

Recent success at targeting immune inhibitory pathways has brought great deal of excitement in the field of cancer therapy. PD-1 is a key receptor expressed on T cells that can mediate immunosuppression by interacting with its ligands PD-L1 and PD-L2 on tumor or antigen presenting cells [21, 22]. Inhibiting PD-1 pathway can enhance anti-tumor activity of cytotoxic T cells. Two blocking antibodies to PD-1, nivolumab and pembrolizumab, have recently been approved by FDA for treating advanced solid tumors including NSCLC. Overall response rate of approximately 20% was achieved in patients with refractory NSCLC [23, 24]. It is important to determine the prognostic and predictive biomarkers that identify patients with high risk of disease progression and patients who will respond to PD-1 inhibitors.

Our finding of high expression of PD-1 on CD4^+^ T cells associates with poor clinical outcome has significant clinical implications as it provides a potential prognostic biomarker for patients with NSCLC. It has been challenging to determine the prognostic value of molecules involved in PD-1 pathway such as PD-L1 and PD-1. Previously published reports have been controversial with some showing a poor prognostic value and others with good or no prognostic significance [16, 17, 18, 19, 25, 26]. The majority of the studies were performed in tumor tissues so that the PD-L1 expression on tumor cells and PD-1 expression on tumors infiltrated by lymphocytes were tested. The challenge is largely due to the small cohorts, limited tissue samples available, and lack of validated experimental methods to quantify the PD-1 or PD-L1 expression in tissues.

In our study, we used peripheral blood samples from patients with NSCLC to demonstrate a negative prognostic value of PD-1 in this population, i.e. high expression of PD-1 associates with poor PFS and OS. It is speculated that high-PD-1 expression in T cells results in immunosuppressed state and less functional for anti-tumor response leading to tumor progression and poor overall clinical outcome. Importantly, we bypassed the need for invasive tissue biopsy, which leads to treatment delays along with a potential risk of complications. Flow cytometry is a defined and well-quantitative method to determine the level of PD-1 expression on blood cells as PD-1 antibodies used in this test has been validated and widely used. Our study provided a highly feasible and effective clinical method to determine the prognosis of patients with NSCLC.

Considerable effort has been placed to identify the predictive biomarker for clinical response to PD-1 pathway treatment. The majority of the studies demonstrated a strong predictive association between tumor surface expressions of PD-L1 and response/outcome. However, it is worthwhile to note that a small portion of patients who are PD-L1 negative do respond to anti-PD-1/anti PD-L1 treatment. In addition, although promising and durable responses were achieved in some patients, others failed to show evidence of activity. In the phase I clinical study testing the safety and efficacy of anit-PD-L1 antibody in patients with advanced cancer, 10% (5/49) objective response and 24% (12/49) stable disease were reported [15]. To identify patients who might benefit from those treatments will be of great clinical impact. In our study, we analyzed PD-1 expression on T cells from peripheral blood of patients receiving anti-PD-L1 treatment. All patients were tested positive for PD-L1. We observed poor clinical outcome in patients with high PD-1 expression at initial diagnosis. This has a significant impact on data providing a potential predictive biomarker for NSCLC patients receiving PD-1/PD-L1 targeted treatment. It should be noted that our data is limited by a small sample size.

In summary, our study demonstrates a prognostic value of PD-1 expression on CD4^+^ T cells from peripheral blood of patients with NSCLC. High PD-1 expression associates with poor clinical outcome. In addition, testing peripheral PD-1 expression has the potential to predict clinical response to treatment of blocking the PD-1/PD-L1 pathway.

## MATERIALS AND METHODS

### Patients

All 42 patients met the clinical radiological and histological criteria for advanced stage (stage IIIB or IV) NSCLC (Table 1). The patients' age ranged from 47 to 82 years old (median 64 years old). There were 19 males and 23 females. Peripheral blood specimens from these patients were obtained, and informed consents were signed for sample collection according to a protocol approved by the Institutional Review Board of Penn State Hershey College of Medicine. Buffy coats from 25 age- and gender-matched normal donors were also obtained from the blood bank of Milton S. Hershey Medicine Center at the College of Medicine. PBMC were isolated by Ficoll-Hypaque gradient separation as described previously [20]. Cell viability was determined by trypan blue exclusion assay with more than 95% viability in all the samples.

### Immunofluorescence staining and flow cytometric analysis

One million PBMCs from patients or normal donors were used to quantify PD-1+% in CD3+CD4+ or CD3+CD8+ T cells by flow cytometry. The following antibodies were used for immunofluorescence staining: PerCP-Cy5.5 conjugated mouse anti-human CD3 (BD Pharmingen, Cat: 560835), FITC conjugated mouse anti-human CD4 (BioLegend, Cat: 317408), APC-Cy7 conjugated mouse anti-human CD8 (BioLegend, Cat: 344714) and PE conjugated mouse anti-human PD-1 (BioLegend, Cat: 329906).

### Statistical analysis

Differences among two groups were statistically analyzed using a two-tailed Student's t test. The Kaplan–Meier survival curves were plotted to evaluate progression free survival (PFS) or overall survival (OS); log-rank (Mantel-Cox) test was used to analyze the statistic difference between the PD-1+% low vs. PD-1+% high patients. Correlation of PD-1 expressed T cells and tumor burden, which was measured following Recist 1.1 guideline, was assessed by Pearson Correlation analysis. A statistically significant difference was reported with p indicated where applicable. Data are reported as the mean ± SE from at least three separate experiments.
